# Current medical support for victims of domestic violence and sexual assault: A nationwide survey among obstetricians and gynecologists in Japan

**DOI:** 10.1111/jog.16272

**Published:** 2025-03-20

**Authors:** Yoshie Kono, Haruyo Atsumi, Kyoko Tanebe, Tomoko Adachi, Satoru Kyo

**Affiliations:** ^1^ Matsue Health Service Center Shimane University Matsue Japan; ^2^ Department of Laboratory Medicine Tokai University School of Medicine Isehara Japan; ^3^ Women's Clinic ‘We TOYAMA’ Toyama Japan; ^4^ Department of Obstetrics and Gynecology Aiiku Maternal and Child Health Center, Aiiku Hospital Tokyo Japan; ^5^ Department of Obstetrics and Gynecology Shimane University Faculty of Medicine Izumo Japan

**Keywords:** domestic violence, Japan, physicians, sex offenses, surveys and questionnaires

## Abstract

**Aim:**

In 2018, one‐stop support centers for victims of sexual crime and violence were established across Japan. Despite this initiative, recent findings suggest inadequate medical response methods for victims of sexual violence. This study conducted a nationwide survey to assess the current state of medical support available to such victims.

**Methods:**

A survey was conducted via e‐mail on 16,500 obstetricians and gynecologists from December 10, 2022, to January 20, 2023. A total of 1387 responses were received (response rate: 8.4%), and 1158 valid responses (valid response rate: 7.0%) were analyzed.

**Results:**

Among the respondents, 76.5% reported examining patients suspected of being victims of domestic or sexual violence, with 90.5% recognizing the definition of sexual violence and 73.8% aware of the one‐stop support centers. However, only 42.3%, 25.9%, and 19.6% had opportunities to learn about sexual violence against children, men, and sexual minorities, respectively. The most common learning opportunity was attending academic lectures on sexual violence. The actual examination rates for cases involving sexual violence against children, men, and sexual minorities were 26.5%, 1.6%, and 1.8%, respectively. Physicians who learned about sexual violence against children, men, and sexual minorities were significantly more likely to have experience in examining each case (*p* < 0.001, *p* < 0.01, *p* < 0.001).

**Conclusions:**

Despite their critical role, obstetricians and gynecologists in Japan have limited opportunities to learn about and support victims of domestic or sexual violence. Urgent actions are needed to ensure timely medical care, establish collaborative practices, and develop systematic protocols nationwide.

## INTRODUCTION

According to a previous survey conducted in Japan, 8.1% of women and 0.7% of men have experienced forced sexual intercourse or similar acts, predominantly during childhood and their 20s.^1^ Approximately 60% of the perpetrators in these cases were spouses or partners, with much of this sexual violence occurring within the context of domestic violence (DV). Victims of sexual violence, including DV, often seek gynecological care because of issues such as unexpected pregnancies, abortions, sexually transmitted infections (STIs), and other complications. In response to these challenges, guidelines for obstetric and gynecological care published by the Japan Society of Obstetrics and Gynecology (JSOG) include protocols for managing female victims of sexual violence.^2^

However, an online survey^3^ examining the prevalence of sexual violence among younger individuals found that 5.1% of males aged 16–24 years had experienced sexual violence involving physical contact, and 2.1% of incidents involving sexual intercourse. Additionally, 32.2% of individuals in the same age group identifying as X gender or nonbinary reported experiencing sexual violence involving physical contact and 12.2% of sexual violence involving intercourse. In Japan, although the situation of overlooked victims, including children, men, and sexual minorities, is gradually becoming clearer, no medical response protocols for these victims have been firmly established.

Given this background, the present study aimed to assess the current awareness and roles of obstetricians and gynecologists nationwide in supporting victims of DV and sexual violence, including women, children, men, and sexual minorities.

## METHODS

### Objective

This study investigated obstetricians and gynecologists working at medical institutions nationwide who were members of the JSOG. With prior approval, a survey questionnaire was distributed to all JSOG members either online or in writing. All participants provided informed consent to participate in the study, and only valid survey responses were included in the analysis.

### Methods

The online survey questionnaire was developed using an online survey system. On December 9, 2022, the survey was announced on the JSOG website, and a survey e‐mail was simultaneously sent to approximately 16,500 registered members. The survey was conducted from December 10, 2022, to January 20, 2023. Additionally, to enhance the response rate, surveys were directly mailed to 5124 obstetricians and gynecologists identified through a nationwide search on the website. Both paper and online surveys included instructions to ensure that the respondents answered only once and did not duplicate their responses.

### Survey items

The survey was composed of items related to the characteristics of the participating obstetricians and gynecologists, their practices in identifying DV and sexual violence during consultations, their knowledge about sexual violence, whether they have had any educational experiences related to sexual violence, and their experiences in providing support to victims of sexual violence. The definition of a child was set as individuals under 15 years old, which aligns with the common criteria for pediatric care in Japan.

### Analysis

Responses were obtained from 1387 participants, resulting in a response rate of 8.4%. After verifying the validity of responses, 1158 valid responses (valid response rate: 7.0%) were included in the analysis. The data supporting the findings of this study are available in the Supporting Information (see Table S1 and Data S1). The results of each survey item were evaluated using simple tabulation and Fisher's exact probability test based on the respondents' characteristics. Statistical analysis was performed using IBM SPSS Statistics 28.0J for Windows (IBM, Armonk, NY, USA) with significance determined at a level of less than 5%.

### Ethical considerations

This study was conducted anonymously with appropriate ethical considerations and was approved by the Medical Research Ethics Committee, Shimane University Faculty of Medicine (Research Management No. KT20221024‐1).

## RESULTS

The characteristics of the surveyed obstetricians and gynecologists are presented in Table 1. Among the valid respondents, 67.7% (781/1154) were Designated Doctors of the Maternal Health Act, with 78.5% (613/781) currently performing induced abortions.

**TABLE 1:** Characteristics of the participants (*n* = 1158).

Characteristics	*N*	Percentage (%)
Age		
≥50 years	734	63.7
<50 years	419	36.2
NA	5	4.0
Gender		
Female doctor	488	42.3
Other than female doctor	666	57.5
NA	4	3.0
Years of practice		
≥21	805	69.7
<21	350	30.2
NA	3	0.3
Hospital type		
University or public emergency‐designated hospital	416	36.0
Other than university or public emergency‐designated hospital	739	63.8
NA	3	0.3
Hospital beds		
≥500	188	16.3
<500	967	83.5
NA	3	0.3
Designation under the Maternal Health Act		
Designated doctor	781	67.7
Other than designated doctor	368	31.8
Prefer not to answer	5	0.4
NA	4	0.3
Induced abortion practice		
Physicians performing induced abortions among designated doctors	613	78.5
Physicians not performing induced abortions among designated doctors	167	21.4
Prefer not to answer	1	0.1
NA	373	0.0

Abbreviation: NA, not available.

Among obstetricians and gynecologists performing induced abortions, 69.3% confirmed the presence of DV and 72.9% confirmed sexual violence at the time of the procedure. The percentages were 60.0% and 62.3%, respectively, when prescribing emergency contraceptive pills (ECPs), and 48.8% and 50.7% when conducting tests for STIs (Table 2).

**TABLE 2:** Confirmation rates of domestic and sexual violence in providing support (%).

Support contents	The type of violence	Confirmed (%)	Not confirmed (%)	Others (%)
Induced abortions	DV (*n* = 614)	69.3	29.0	1.8
	SV (*n* = 561)	72.9	25.5	1.6
Emergency contraceptive pills	DV (*n* = 1151)	60.0	34.9	5.1
	SV (*n* = 1147)	62.3	32.7	5.0
Sexually transmitted infection tests	DV (*n* = 1150)	48.8	49.4	1.8
	SV (*n* = 1150)	50.7	47.5	1.8

Abbreviations: DV, domestic violence; SV, sexual violence.

In addition, 90.5% of the obstetricians and gynecologists reported knowing the precise definition of sexual violence, 73.8% were aware of the existence of one‐stop support centers for sexual crime and violence victims (one‐stop centers), and 76.5% had examined patients suspected of being victims of DV or sexual violence. Regarding knowledge acquisition related to sexual violence, 78.8% of the respondents had encountered information on sexual violence in some form (Table 3).

**TABLE 3:** Physicians' knowledge of domestic and sexual violence (*n* = 1158).

Category	Question item	*N*	Percentage (%)
Knowledge of sexual violence	Know the precise definition of sexual violence	1044	90.5
	Do not know the precise definition of sexual violence	109	9.4
	NA	5	0.4
Aware of one‐stop centers	Aware of one‐stop centers	847	73.8
	Not aware of one‐stop centers, Other	300	25.9
	NA	6	0.5
Experience with victims	Have been involved in the examination of patients suspected of being victims of domestic and sexual violence	878	76.5
	Have not been involved in the examination of patients suspected of being victims of domestic and sexual violence	242	20.9
	Prefer not to answer, Other	28	2.4
	NA	10	0.9
Training and resources	Encountered information on sexual violence in some form	908	78.8
	Have not encountered information	244	21.1
	NA	6	0.5

*Note*: One‐stop centers refer to one‐stop support centers for victims of sexual crimes and violence.

Abbreviation: NA, not available.

When asked about learning opportunities related to sexual violence against children, men, and sexual minorities, the percentages were 42.3%, 25.9%, and 19.6%, respectively, which were lower than those related to all sexual violence. Correspondingly, the percentages of obstetricians and gynecologists who had examined such patients were 26.5%, 1.6%, and 1.8%, respectively, reflecting the predominantly female patient population in obstetrics and gynecology (Table 4).

**TABLE 4:** Physicians' learning opportunities and examination experience related to domestic and sexual violence (*n* = 1158).

Category	Question item	*N*	Percentage (%)
Learning opportunities	Opportunity to learn about sexual violence against children	484	42.3
	No opportunity to learn about sexual violence against children	660	57.0
	NA	14	1.2
	Opportunity to learn about sexual violence against men	296	25.9
	No opportunity to learn about sexual violence against men	847	73.1
	NA	15	1.3
	Opportunity to learn about sexual violence against sexual minorities	224	19.6
	No opportunity to learn about sexual violence against sexual minorities	919	79.4
	NA	15	1.3
Examination experience	Examination experience for child victims	298	26.5
	No examination experience for child victims	828	71.5
	NA	32	2.8
	Examination experience for male victims	18	1.6
	No examination experience for male victims	1105	98.4
	NA	35	3.0
	Examination experience for sexual minority victims	20	1.8
	No examination experience for sexual minority victims	1097	94.7
	NA	41	3.5

Abbreviation: NA, not available.

Regarding knowledge acquisition related to sexual violence, the most common sources were the mass media and academic conferences. Only 8.6% of the obstetricians and gynecologists had attended lectures on sexual violence in medical school, where there were few opportunities to learn about children, men, and sexual minorities (Table 5).

**TABLE 5:** Sources of information and educational experience related to sexual violence (*n* = 1158, multiple answers, %).

Source	Received SV info	Learn about SV against children	Learn about SV against men	Learn about SV against sexual minorities
Mass media	57.1	20.1	13.3	8.9
Academic lectures	44.6	27.3	13.9	10.9
Internet	39.5	15.0	10.8	8.3
Magazines and/or books	23.5	10.9	6.7	5.6
Lectures in medical universities	8.6	3.6	1.5	0.9
Citizen public lectures	4.4	3.5	1.6	0.9
Others	8.0	4.7	2.3	1.9

Abbreviation: SV, sexual violence.

A further examination of the correlation between the experience of examining victims of sexual violence and the characteristics of obstetricians and gynecologists revealed several significant findings (Table 6). Female doctors (*p* < 0.001) and those working in university hospitals or emergency‐designated hospitals (*p* = 0.049) showed significantly higher rates of examination experience for child victims. Additionally, characteristics such as knowing the definition of sexual violence (*p* < 0.001), encountering information on sexual violence (*p* < 0.001), awareness of one‐stop support centers (*p* < 0.001), and having had opportunities to learn about sexual violence against children (*p* < 0.001), men (*p* < 0.001), and sexual minorities (*p* < 0.001) were associated with significantly higher rates of examination experience for child victims.

**TABLE 6:** Correlation between physician attributes and examination experience regarding victims of sexual violence (%).

Item	Children			Men			Sexual minorities		
	Examination experience (*n* = 298)	No experience (*n* = 828)	*P*‐value	Examination experience (*n* = 18)	No experience (*n* = 1105)	*P*‐value	Examination experience (*n* = 20)	No experience (*n* = 1097)	*P*‐value
Age ≥50 years	66.3	62.9	0.292	77.8	63.5	0.323	80.0	63.2	0.160
Female doctor	62.3	35.0	<0.001	55.6	42.1	0.337	55.0	42.2	0.262
Years of practice ≥21	68.1	70.2	0.509	88.9	69.2	0.117	85.0	69.1	0.148
Works at a university or designated public emergency hospital	40.9	34.4	0.049	27.8	36.2	0.622	25.0	36.4	0.354
Hospital beds ≥500	18.5	15.5	0.234	22.2	16.2	0.516	0.0	16.6	0.060
Designated Doctor of Maternal Health Act	68.8	67.6	0.682	72.2	67.8	0.819	85.0	67.6	0.219
Knows the definition of sexual violence	95.6	88.9	<0.001	94.4	90.6	1.000	95.0	90.7	1.000
Experience of encountering information on sexual violence	90.9	74.3	<0.001	94.4	78.4	0.144	100.0	78.2	0.012
Awareness of one‐stop centers	82.2	71.0	<0.001	83.3	73.7	0.431	90.0	73.6	0.124
Opportunity to learn about sexual violence against children	63.9	34.9	<0.001	52.9	42.2	0.460	80.0	41.8	<0.001
Opportunity to learn about sexual violence against men	43.9	19.5	<0.001	58.8	25.3	0.004	75.0	25.1	<0.001
Opportunity to learn about sexual violence against sexual minorities	35.3	14.0	<0.001	47.1	19.3	0.009	75.0	18.8	<0.001

*Note*: One‐stop centers refer to one‐stop support centers for victims of sexual crimes and violence.

According to Fisher's exact test, for male victims, those who had opportunities to learn about sexual violence against men (*p* = 0.004) and sexual minorities (*p* = 0.009) had significantly higher rates of examination experience. Regarding sexual minority victims, those who had encountered information on sexual violence (*p* = 0.012) and had opportunities to learn about sexual violence against children (*p* < 0.001), men (*p* < 0.001), and sexual minorities (*p* < 0.001) also had significantly higher rates of examination experience.

## DISCUSSION

In Japan, amendments to the Penal Code in 2017 and 2023 expanded the definition of rape and forcible sexual intercourse beyond vaginal penetration to include anal and oral intercourse, thereby encompassing men and sexual minorities as victims. Furthermore, in 2024, revisions to the DV Prevention Act broadened the definition of DV from solely “physical violence” to encompass situations where there is a significant risk of serious life, body, freedom, honor, or property harm through threats. There is now an increased expectation for physicians to provide objective evidence of DV.^2^

In collaboration with the JSOG, we conducted an unprecedented nationwide survey related to the response to victims of sexual violence. It has been reported that victims of DV and sexual violence frequently visit obstetrics and gynecology departments for purposes such as emergency contraception, testing for STIs, pregnancy diagnosis, and induced abortion.^2^, ^4^ The results showed that the proportion of obstetricians and gynecologists confirming the presence of DV or sexual violence during consultations was approximately 70% during induced abortions, 60% during emergency contraception prescriptions, and 50% during testing for STIs.

Adequate responses to DV and sexual violence represent significant medical and social challenges, with potentially serious consequences for physical and mental health if not properly addressed.^5^, ^6^ The timely recognition of violence during consultations can lead to substantial changes in treatment and responses, thereby potentially preventing further violence and worsening symptoms. Since the 1980s, the American College of Obstetricians and Gynecologists has recommended regular DV screening for all women, recognizing its significant impact on women's health.^7^ A study by Horan et al. in 1998 reported that 30% of obstetricians and gynecologists had received training on DV during medical school, with 67% pursuing ongoing education afterward. Despite this, only about 30% actively performed DV screening in practice.^8^ Similarly, a 2020 study by Jones et al. found that less than half of obstetricians and gynecologists regularly conducted screenings.^9^

A 2021 survey of 16,680 obstetricians and gynecologists conducted by the Japan Association of Obstetricians and Gynecologists (5249 valid responses; 31.5% valid response rate) found that 39.4% of physicians check whether violence has occurred when prescribing ECPs.^10^ Our results were higher than we had expected, which may be the result of a bias from the low response rates. In the future, efforts to inquire actively about DV and sexual violence during consultations with patients and their families will be required in the field of obstetrics and gynecology.

Regarding questions related to the participants' knowledge and experience of sexual violence, it was estimated that the main sources of information were the mass media and academic lectures, and few systematic learning opportunities were available. Among active obstetricians and gynecologists, only 8.6% reported receiving lectures related to sexual violence during their medical education at university, highlighting limited learning opportunities, particularly regarding sexual violence against children, men, and sexual minorities.

Additionally, 26.5% of obstetricians and gynecologists reported having experience in treating child victims of sexual violence. Children who had been victims of sexual violence were more likely to be examined by a female doctor working in a large hospital. In examinations of victims of sexual violence, detailed charting and specimen collection for evidence are required, in addition to wound and infection examinations.^11^ Especially concerning child victims, conducting forensic interviews is crucial, and referrals to specialist physicians may be necessary.^12^ Establishing medical institutions where child victims can promptly seek medical support and ensuring collaboration and appropriate responses are urgently needed.

Given the inherent focus of obstetrics and gynecology on female patients, a limited number of physicians reported having experience in supporting male and sexual minority victims of sexual violence in this survey. While the evaluation of physicians who have experience in treating male and sexual minority victims of sexual violence was limited because of their small numbers, statistically significant trends were observed in physicians who had learning opportunities related to sexual violence against children, men, and sexual minorities.

Regarding previously unidentified victims such as males and sexual minorities, it remains difficult to determine which medical departments within health‐care institutions are capable of providing appropriate care. Potential departments include emergency medicine, urology, pediatrics, gastroenterology, surgery, otolaryngology, oral surgery, psychiatry, and psychosomatic medicine. It has been reported in Western countries that victims of sexual violence, who often present with injuries affecting multiple areas of the body, are also seen in emergency care centers^13^ and by general practitioners (GPs).^14^ In the future, it will be necessary to establish a framework for acquiring basic knowledge on sexual violence among all physicians, not limited to specific medical departments such as obstetrics and gynecology, beginning with medical education for medical students and trainees.

This study does have some limitations. First, the response rate to the survey was insufficient. Although we anticipated a low response rate, we made efforts to increase it by not only sending e‐mail surveys through the JSOG mailing list, but also directly sending survey request letters to major medical institutions. Despite these efforts, the valid response rate was only 7.0%. Therefore, it is highly likely that the responses came mainly from physicians who had an interest in supporting victims of sexual violence, introducing a potential bias. Further surveys that can provide a more accurate reflection of reality are needed, as well as the development of more effective interventions.

## AUTHOR CONTRIBUTIONS

**Yoshie Kono:** Conceptualization; funding acquisition; methodology; project administration; resources; supervision; validation; writing – original draft; writing – review and editing. **Haruyo Atsumi:** Formal analysis; investigation; methodology; validation. **Kyoko Tanebe:** Conceptualization; methodology; validation. **Tomoko Adachi:** Conceptualization; methodology; validation. **Satoru Kyo:** Conceptualization; methodology; validation.

## CONFLICT OF INTEREST STATEMENT

The authors declare no conflicts of interest.

## Supporting information


**Table S1.** Questionnaire—The study data.

**Data S1.** Supplementary data: Variable information in SPSS.

## Data Availability

The data that supports the findings of this study are available in the supplementary material of this article.
